# HCV screening, linkage to care, and treatment patterns at different sites across one academic medical center

**DOI:** 10.1371/journal.pone.0218388

**Published:** 2019-07-10

**Authors:** Paul Calner, Heather Sperring, Glorimar Ruiz-Mercado, Nancy S. Miller, Chris Andry, Leandra Battisti, Katy Scrudder, Fiona Shea, Angelica Chan, Elissa M. Schechter-Perkins

**Affiliations:** 1 Department of Emergency Medicine, Boston University Medical Center, Boston, Massachusetts, United States of America; 2 Boston University Master’s Program in Public Health, Section of Infectious Disease, Department of General Internal Medicine, Boston University Medical Center, Boston, Massachusetts, United States of America; 3 Center for Infectious Diseases and Public Health Programs Section of Infectious Diseases, Department of General Internal Medicine Boston University Medical Center, Boston, Massachusetts, United States of America; 4 Clinical Microbiology & Molecular Diagnostics Laboratory Medicine, Boston University Medical Center, Department of Pathology & Laboratory Medicine, Boston, Massachusetts United States of America; 5 Department of Pharmacy Operations & Project Management, Boston Medical Center, Boston, Massachusetts, United States of America; University of Cincinnati College of Medicine, UNITED STATES

## Abstract

**Background:**

It is unclear whether sites that screen large numbers of patients for Hepatitis C Virus but achieve limited follow-up are more or less effective at having patients succeed through linkage and treatment than lower volume sites that have higher linkage percentages. The objective was to compare the rates of HCV identification, linkage to care, and treatment success between different study sites including the Emergency Department, 3 outpatient clinics with unique patients, and the inpatient setting at one medical center

**Methods:**

This is a descriptive analysis of 2 years of data from a protocol that integrated HCV screening and treatment into clinical services throughout multiple departments in one medical center. The program used a best practice advisory to prompt testing at all sites, with different triggers for it to fire at each site, and one central navigation program that attempted to link all patients diagnosed with hepatitis C virus to outpatient care. Outcomes included volume of tests performed in each site, Antibody and RNA rates at each site, demographic data, navigation and linkage outcomes, and post-linkage treatment completion.

**Results:**

28,435 patients were screened across 5 clinical locations. RNA+ rates and absolute numbers linked to MD (linkage rates among all RNA+) were: ED 7.2% RNA+, 224 (22.6%) linked; Inpatient 14.8% RNA+, 27 (17.6%) linked, General Internal Medicine 3.9% RNA+, 269 (65.8%) linked, Infectious Diseases 4.0% RNA+, 34(70.8%) linked, Family Medicine 2.0% RNA+, 28 (75.7%) linked. Demographics, linkage barriers, and treatment initiation rates were different at all sites.

**Conclusion:**

Among sites there were differences in the sociodemographic characteristics of patients diagnosed with HCV, as well as differences in the success linking patients to outpatient care. At this medical center, the ED screened the most patients, the inpatient area had the highest RNA positivity rate, the FM clinic had the highest linkage rate, GIM linked the most patients by absolute number, and GIM also had the highest number of patients start treatment.

## Introduction

Hepatitis C Virus (HCV) infection has been found to be highly prevalent in the United States, with an estimated 3.5 million people suffering from the disease[1–3]. Patients with chronic HCV infection are often asymptomatic and approximately 50% of patients in the United States with chronic HCV are unaware of their infection[3]. Several current treatments for HCV are considered curative[4,5] and curing HCV results in decreased transmission, and decreased morbidity and mortality[6,7]. Therefore, both the U.S. Centers for Disease Control and Prevention (CDC) and the U.S. Preventive Services Task Force (USPSTF) endorsed guidance for routine one-time HCV Antibody (Ab) screening for individuals born from 1945 to 1965 (the “birth cohort”) as well as continued targeted testing for injection drug users (IDUs) and others at high risk for HCV infection[8].

However, there are multiple barriers impeding diagnosis and successful treatment. The cascade of care from diagnosis through cure has been described in the literature[9–11] and is a useful tool in measuring where patients fall out of care. The cascade includes making the initial diagnosis, confirming the diagnosis, disclosing the diagnosis to the patient, and linking the patient to outpatient follow up care for disease staging, treatment initiation, treatment completion, and testing for sustained virologic response (SVR).

Determining the best sites to identify infected patients while simultaneously successfully linking them to treatment has a significant impact on program efficacy and its cost-effectiveness. It is unclear whether sites that screen or diagnose large numbers of patients with limited follow-up are ultimately more or less effective at having high numbers of patients succeed through the linkage and treatment cascade to eventually achieve SVR than lower volume sites that have higher linkage percentages. There is little empirical data comparing the efficacy of testing patients in different settings at one large medical center.

The authors developed an HCV screening and treatment program at their institution that was implemented in November 2016. The objective for this study was to compare the rates of HCV identification, LTC, treatment completion, and SVR between different study sites including the Emergency Department (ED), 3 outpatient clinics, and the inpatient setting.

## Methods

### Study design

This is a descriptive analysis of the data from a protocol that integrated HCV screening and treatment into clinical services throughout multiple departments in one medical center. The outcomes of the first two years of HCV screening, LTC efforts, and treatment success are reported. This project was approved by the Institutional Review Board at Boston University Medical Center. This study adhered to the Strengthening the Reporting of Observational Studies in Epidemiology (STROBE) guidelines for cohort studies.

### Study setting and population

Boston Medical Center (BMC) is an urban, academic facility that receives approximately 1,150,000 patient visits per year. The medical center is recognized as the primary “safety net” provider of care for the city’s indigent and most vulnerable population. In the medical center, more than 70% of patients identify as minority; more than 50% identify as African American; and more than 20% identify as Hispanic/Latino. Approximately 25% of BMC patients are homeless. Outpatient sites in this study included clinics in General Internal Medicine (GIM) which cares for approximately 38,000 patients/year, Family Medicine (FM) which cares for 8,800 patients per year across all ages, and The Center for Infectious Diseases (CID), which cares for approximately 1,600 adult and pediatric patients in a year, with a focus on HIV/AIDS and STDs. Each of these outpatient sites has a unique mission and focus, and a patient can obtain his/her primary care in only one of the clinics. The Emergency Department was another site studied, which has over 133,000 visits annually, 48% are by patients who don’t identify a primary care provider and therefore don’t routinely seek outpatient care. The inpatient service was the final study site, and the institution has approximately 26,000 adult and pediatric admissions annually.

### HCV screening and linkage to care program

The objective of the HCV program was to increase diagnosis of HCV and LTC in the institution. The first step was to implement hospital-wide reflex testing for HCV RNA and genotyping for all specimens identified as HCV Ab seropositive. The HCV screening program, as it was first implemented in the ED, is described in detail elsewhere[1] and the other sites in the medical center followed a similar procedure, with a few key differences.

The overall method for augmenting screening throughout the medical center was the utilization of a multi-purpose Best Practice Advisory (BPA) that fired to alert the provider that a patient was eligible for testing, and generated a laboratory order if the provider chose “Yes” in response to the BPA prompt. At all sites, prior to firing, the BPA did a “look-back” at the EMR in the prior year, and excluded those who had a complete HCV panel result in the EMR in the prior 6 months. This ensured that patients who were tested at one site and then presented to another site were not re-tested. Each site had differences in the eligibility criteria (ie trigger for the BPA to fire) based on the feedback of the physician stakeholders at each site. In the ED, opt-out HCV screening was offered (through BPA support) to all ED patients over 13 years of age who were undergoing phlebotomy for any clinical purpose. In the outpatient clinics, HCV screening was offered, with BPA support, to all patients who met high risk criteria (born between 1945 and 1965 or documented history of IDU). Patients admitted to the inpatient wards (Inpatient) were tested through provider discretion, without the decision-support of a BPA. At the time of testing, efforts were made by registration staff and by the providers in each location to ensure proper contact information. Implementation of the BPA was monitored by the research team.

Due to previously identified challenges in linking HCV patients to care at this institution, augmented linkage support was provided to supplement the routine care coordination services offered by the outpatient sites. Linkage services across all 5 HCV testing sites were managed through a single, standardized linkage to care program that was supported by a part-time data analyst and two full-time public health navigators who enrolled HCV Ab-positive patients into navigation. Patients were given a unique identifier and could not be enrolled into linkage services through more than one testing site. This system ensured that patients across all sites received the same linkage efforts and followed the same linkage protocol.

Navigators contacted patients found to be positive for HCV RNA, either immediately if patients were diagnosed in the ED/inpatient, or after 2 weeks if patients were diagnosed in a clinic setting (in order to give their primary care provider an opportunity to contact them first). Patients with equivocal results or those whose tests failed to reflex were also contacted to facilitate repeat testing. Patients not considered to have a need for linkage included: 1) patients receiving primary care at an outside medical system known to treat HCV, 2) incarcerated persons, 3) those terminally ill or deceased since testing, 4) pregnant women (until after pregnancy has ended), 5) individuals with an HCV viral load < 500 IU/mL (this was a decision made by the HCV treatment providers, prior to program initiation), 6) those with EMR notes documenting a physician management decision not to treat at that time, and 7) those with no contact information in the EMR. The remainder of patients were considered to have a need for linkage.

Patients that did not have health insurance were directed to institutional services that helped them obtain it (the medical center is able to obtain same-day-coverage to bridge the gap before Medicaid begins) and then patients were linked to care. The site they were linked to was one of three sites that treat HCV positive patients at the institution, which included GIM, CID, and gastroenterology (GI), and was based on an algorithm developed by HCV treatment providers. Once patients were linked to care, their progress through the treatment cascade was supported by their physicians and hospital case managers.

### Data collection

Data was collected prospectively beginning on July 1, 2016. Testing data was collected through July 31, 2018, and linkage and treatment data were collected through September 20, 2018. A relational database was constructed for tracking patients in the program and for data analysis using Salesforce healthcloud (Salesforce.com). All hospital HCV Ab tests were downloaded daily and organized by testing site. Results from patients under 13 years, those tested in a non-study site, and those previously enrolled in our navigation program were removed. The remaining patients were assigned study IDs and enrolled into Salesforce. The navigators tracked these patients’ charts and updated Salesforce with RNA results once received. The navigators and data analyst populated the database using electronic queries of the EMR, manual chart reviews, and direct communications with patients. Data elements abstracted from the EMR included age, sex, race/ethnicity, marital status, residence, previous HCV test results, and HIV and Hepatitis B co-infections. Study staff performed manual chart reviews to extract histories of substance use based both on problem lists and reviews of clinical encounter notes. Navigators also communicated directly with patients to ask about IDU history using a formal script. Manual chart reviews were conducted to determine current homelessness based on clinical support notes, problem lists, and current address (ex. homeless shelters). Post-linkage data was collected and entered by case managers.

All data abstraction was validated through the use of multiple navigators that verified data for each patient throughout the study period. Any data discrepancies went to the study PI for immediate review and correction.

### Outcome measures and data analysis

The first outcome is a report of the total number of unique HCV Ab tests performed by date and location. Equivocal HCV Ab test results were defined as those results meeting “gray zone” criteria per the assay manufacturer’s instructions. Ab prevalence was defined as the number of positive Ab test results among all tests with positive results. RNA positive patients are reported as those with a detectable HCV viral load of >500 IU/mL. New diagnosis was defined as no EMR record of prior positive HCV Ab. A linkage to an HCV intake was defined as any RNA+ patient who had a need for linkage and who attended an appointment with an MD/DO or midlevel provider that is able to treat HCV, and where HCV was discussed on or before 9/20/2018. Linkage success is described both as a proportion of those considered to have a need for linkage as well as a proportion of all patients found to be RNA+. Among reasons not linked, “Unreachable/ Lost to Follow-Up” was defined as those who were not reachable by navigators following a sequence of four outreach phone calls, a call to the emergency contact, and a mailed letter, either with no initial contact, or starting from the last contact made where a linkage was not successfully completed. Post-intake, SVR is defined as those patients with a post-treatment test showing an undetectable viral load at ≤500 IU/L at least 12 weeks after treatment completion.

Categorical variables were described using patient counts and percentages, while continuous variables were described using mean and standard deviation. Outcomes were measured as binary variables, where patients either did or did not move on to the next step in the cascade of care.

## Results

Utilization of the BPA was monitored by the research team and was similar at each of the sites based on qualitative feedback from the Information Technology department and the clinic medical directors.

Table 1 shows the demographic characteristics and co-morbid conditions among patients that were screened and those that screened positive for HCV RNA at each individual site. Table 2 shows the linkage characteristics among those RNA+ across each testing site. This table also shows the reasons that patients dropped off of the treatment cascade at key junctures. Major findings included: substantial differences in RNA+ rates at different sites with the ED and inpatient having the highest rates, substantial differences in homeless rates at different sites with the ED and inpatient having the highest rates, substantial differences in linkage eligibility among the sites with the ED and inpatient having fewer patients with a need for linkage, and substantial differences in actual linkages and treatment starts, with the ED and inpatient having the lowest linkage/treatment rates.

**Table 1:** Sociodemographic characteristics by testing site, from July 1 2016 through July 31, 2018.

Variable	ED	GIM	Inpatient	CID	FM
**Number of HCV Tests Performed by Date/Location**					
*2016 (July 1- December 31)*	2598 (18.8%)	2289 (21.7%)	-	-	239 (13.1%)
*2017 (January 1- December 31)*	8070 (58.4%)	5580 (52.9%)	506 (49.1%)	731 (60.9%)	1100 (60.1%)
*2018 (January 1- July 31)*	3161 (22.9%)	2677 (25.4%)	525 (50.9%)	469 (39.1%)	490 (26.8%)
**Total Unique Ab Tests Performed**	13,829	10,546	1,031	1,200	1,829
**Antibody +**	1806 (13.1%)	769 (7.3%)	273(26.6%)	109 (9.1%)	90 (4.9%)
**Ab Test Indeterminate**	53 (0.004%)	39 (0.004%)	3 (0.003%)	5 (0.004%)	10 (0.005%)
**RNA Tests Conducted** *(percent of Ab+)*	1767 (97.8%)	762 (99.1%)	264 (96.7%)	108 (99.1%)	90 (100.0%)
**RNA Positive** *(percent of Ab+)*	990 (56.0%)	409 (53.2%)	153 (56.0%)	48 (44.0%)	37 (41.1%)
**RNA Positive** (*percent of Ab performed)*	990 (7.2%)	409 (3.9%)	153 (14.8%)	48 (4.0%)	37 (2.0%)
**Newly Diagnosed HCV Cases** *(percent of Ab+)*	493 (49.8%)	244 (59.7%)	99 (64.7%)	21 (43.8%)	19 (51.4%)
**Male**	677 (68.4%)	303 (74.1%)	85 (55.6%)	31 (64.6%)	24 (64.9%)
**In Baby Boomer Birth Cohort (1945–1965)**	361 (36.5%)	133 (32.5%)	44 (28.8%)	17 (35.4%)	25 (67.6%)
**Average Age** (SD)	46.2 (14.1)	44.1 (13.2)	42.2 (14.8)	44.2 (13.4)	52.9 (12.3)
**Age Distribution**					
*18–25*	33 (3.3%)	13 (3.2%)	11 (7.2%)	3 (6.3%)	2 (5.4%)
*26–35*	231 (23.3%)	126 (30.8%)	63 (41.2%)	12 (25.0%)	3 (8.1%)
*36–45*	219 (22.12%)	89 (21.8%)	23 (15.0%)	10 (20.8%)	2 (5.4%)
*46–55*	235 (23.7%)	75 (18.3%)	17 (11.1%)	9 (18.8%)	9 (24.3%)
*56–65*	211 (21.1%)	79 (19.3%)	27 (17.7%)	13 (27.1%)	18 (48.7%)
*66–75*	43 (4.3%)	26 (6.4%)	11 (7.2%)	1 (2.1%)	3 (8.1%)
*≥76*	18 (1.8%)	1 (0.2%)	1 (0.7%)	0 (0.0%)	0 (0.0%)
**Race**					
*American Indian/ Alaskan Native*	4 (0.4%)	1 (0.2%)	0 (0.0%)	1 (2.1%)	1 (2.7%)
*Asian*	12 (1.2%)	8 (2.0%)	0 (0.0%)	0 (0.0%)	0 (0.0%)
*Black/ African American*	299 (30.2%)	113 (27.6%)	35 (22.9%)	13 (27.1%)	21 (56.8%)
*Native Hawaiian/ Pacific Islander*	0 (0.0%)	1 (0.2%)	0 (0.0%)	0 (0.0%)	0 (0.0%)
*White*	531 (53.6%)	235 (57.5%)	91 (59.5%)	26 (54.2%)	10 (27.0%)
*Declined/ Unknown*	144 (14.6%)	51 (12.5%)	27 (17.7%)	8 (16.7%)	5 (13.5%)
**Ethnicity** *(# Identifying as Hispanic)*	177 (17.9%)	50 (12.2%)	24 (15.7%)	10 (20.8%)	3 (8.1%)
**Marital Status**					
*Divorced*	68 (6.9%)	29 (7.1%)	11 (7.2%)	1 (2.1%)	5 (13.5%)
*Legally Separated*	41 (4.1%)	12 (2.9%)	3 (2.0%)	2 (4.2%)	3 (8.1%)
*Married*	73 (7.4%)	44 (10.8%)	10 (6.5%)	2(4.2%)	6 (16.2%)
*Other*	19 (1.9%)	12 (2.9%)	6 (3.9%)	0 (0.0%)	1 (2.7%)
*Single*	759 (76.7%)	307 (75.1%)	120 (78.4%)	41 (85.4%)	21 (56.8%)
*Widowed*	30 (3.0%)	5 (1.2%)	3 (2.0%)	2 (4.2%)	1 (2.7%)
**Residence** *(inside Boston)*	673 (68.0%)	266 (65.0%)	75 (49.0%)	28 (58.3%)	28 (75.7%)
**Hep B Co-infection**	49 (5.0%)	10 (2.4%)	4(2.6%)	2 (4.2%)	0 (0.0%)
**HIV Co-Infection**	73 (7.4%)	6 (1.5%)	9 (5.9%)	22 (45.8%)	1 (2.7%)
**Homeless**	689 (69.6%)	199 (48.7%)	98 (64.1%)	27 (56.3%)	15 (40.5%)
**Ever IDU**	768 (77.6%)	326 (79.7%)	129 (84.3%)	43 (89.6%)	19 (51.4%)

**Table 2:** Linkage and treatment results by testing site, from July 1, 2016 through July 31, 2018.

Variable	ED	GIM	Inpatient	CID	FM
**Linkage to Care**					
**Total RNA+**	990	409	153	48	37
**Need for Linkage Intake**	682 (68.9%)	390 (95.4%)	116 (75.8%)	44 (91.7%)	35 (94.6%)
**Reasons not needing linkage**					
*Receiving HCV care elsewhere*	214 (69.5%)	17 (89.5%)	12 (32.4%)	4 (100%)	1 (50.0%)
*Terminally Ill or Deceased*	45 (14.6%)	1 (5.3%)	20 (54.1%)	0 (0.0%)	0 (0.0%)
*Incarcerated*	49 (15.9%)	1 (5.3%)	5 (13.5%)	0 (0.0%)	1 (50.0%)
**Linked to MD Intake Appt** (Among all RNA +)	224 (22.6%)	269 (65.8%)	27 (17.6%)	34 (70.8%)	28 (75.7%)
**Linked to MD Intake Appt** (Among "Need for linkage")	224 (32.8%)	269 (69.0%)	27 (23.3%)	34 (77.3%)	28 (80.0%)
**Reasons not linked**					
*No contact information*	140 (30.6%)	19 (15.8%)	23 (26.1%)	0 (0.0%)	0 (0.0%)
*Refused to be linked to care*	50 (10.9%)	13 (10.8%)	10 (11.4%)	2 (20.0%)	2 (28.6%)
*Unreachable—Lost to follow-up*	219 (47.8%)	64 (53.3%)	28 (31.8%)	2 (20.0%)	2 (28.6%)
*In Progress*	49 (10.7%)	24 (20.0%)	27 (30.7%)	6 (60.0%)	3 (42.9%)
**TREATMENT PROGRESS OF RNA+**					
**Treatment Started** *(Among all RNA+)*	87/990 (8.9%)	145/409 (35.5%)	10/153 (6.5%)	11/48 (22.9%)	22/37 (59.5%)
**Treatment Completed** *(Among all RNA+)*	58/990 (5.9%)	111/409 (27.1%)	4/153 (2.6%)	11/48 (22.9%)	15/37 (40.5%)
**Achieved SVR** *(Among all RNA+)*	42/990 (4.2%)	84/409 (20.5%)	2/153 (1.3%)	7/48 (14.6%)	12/37 (32.4%)
**TREATMENT PROGRESS OF "LINKED"**					
**Treatment Started** *(Among All "Linked")*	87/224 (38.8%)	145/269 (53.9%)	10/27 (37.0%)	11/34 (32.4%)	22/28 (78.6%)
**Treatment Completed** *(Among All "Linked")*	58/224 (25.9%)	111/269 (41.3%)	4/27 (14.8%)	11/34 (32.4%)	15/28 (53.6%)
**Achieved SVR** *(Among All "Linked")*	42/224 (18.8%)	84/269 (31.2%)	2/27 7.4%)	7/34 (20.6%)	12/28 (42.9%)

**Fig 1:**
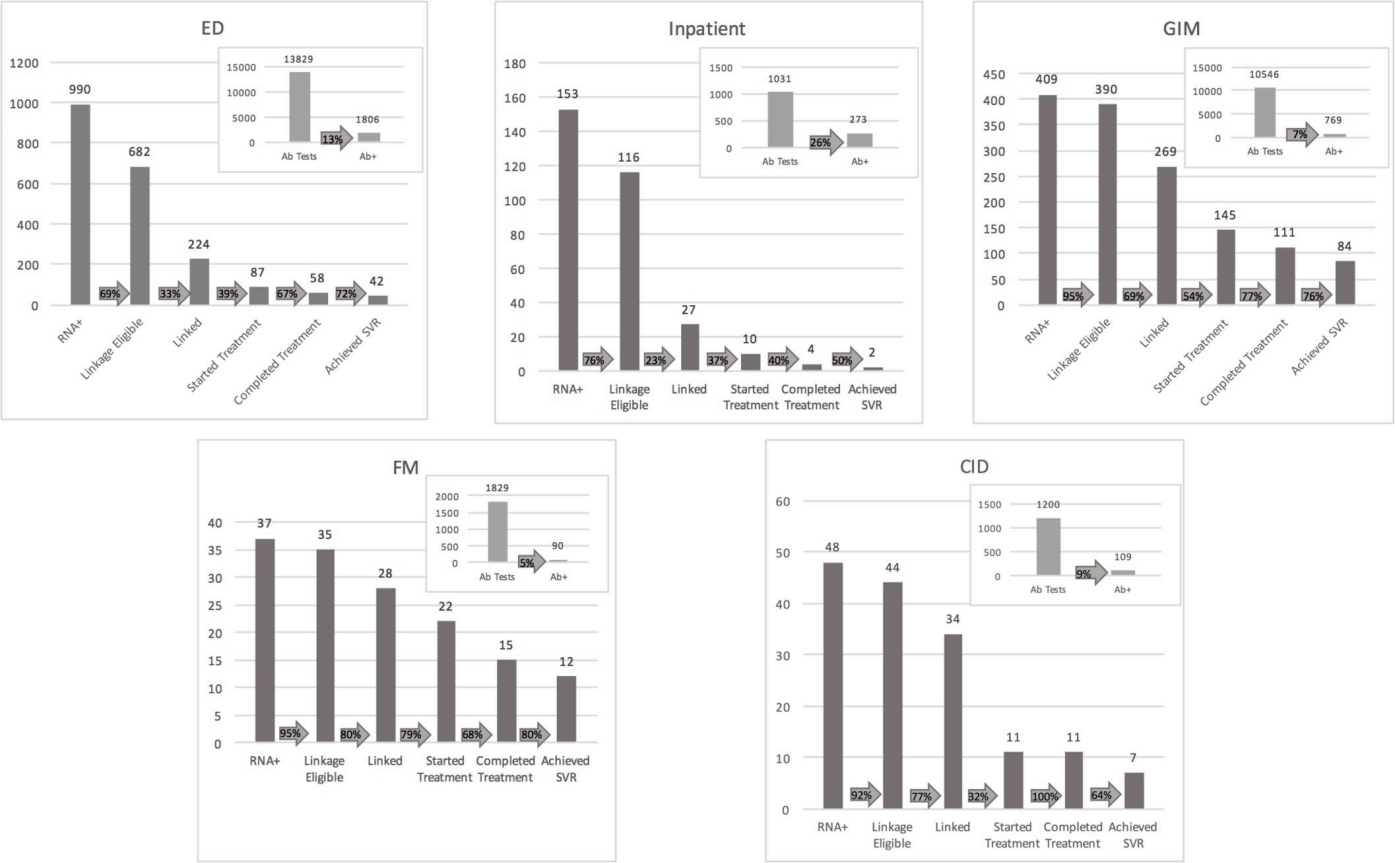
Linkage to care progress by site.

Fig 1 shows progress along successive stages of the HCV treatment cascade as patients progressed from disease diagnosis through cure (SVR), for each individual study site. There are major differences in where significant drop-offs occurred, depending on site.

## Discussion

This study reports on the evaluation of over 1,600 patients diagnosed with HCV, and reveals many significant differences in patient populations across five medical center testing sites, as well as large differences in HCV positivity rate, need for linkage, linkage to care rates, and treatment success. It found that the ED and inpatient sites demonstrated significantly lower rates of linkage to HCV care compared to the outpatient sites (GIM, FM, and CID). Notably, they also had the lowest proportion of “need for linkage” patients. Although the inpatient wards had fewer patients tested, it had a similar profile to the ED, with a high rate of disease but a low linkage percentage. The outpatient settings tested a somewhat lower volume of patients and saw a lower disease rate. However, they had considerably more success in linking patients to care.

Other recent studies have investigated linkage success from different testing sites, including MacLean et al who had a linkage success rate of 90.1% (164/182) of patients screened in a primary care setting [12], Goel et al who found 60 of the 84 (71%) patients newly identified in outpatient primary care clinics were linked to care [13], Zuckerman et al who found 120/187 (64%) of patients screened in an infectious disease completed evaluation for treatment [14], and Anderson et al who found 97/301 (32%) of patients identified in an ED attended their follow-up appointment [15]. The data presented here are consistent with the rates above for both the ambulatory and the ED sites.

In the cascade of care literature there are several factors which have shown to result in poor linkage to HCV care. These include: the type of insurance a patient has, the health system the patient is operating within, incorrect follow up contact information or no contact information available, homelessness, psychiatric disease, physical illnesses preventing patients from traveling to follow up, and substance abuse[16–19]. Across the five testing sites in this cohort, even though they are patients within the same medical center, there are significant differences in key patient population characteristics, many of which fall into this list. The patients tested and enrolled from each of these sites are significantly different regarding race, ethnicity, age (and % of “baby boomers”), marital status, HBV and HIV coinfections, homelessness, and history of IDU. Despite all these patients presenting within the same hospital system, there were stark differences in the demographic stratifications based on where they presented and were tested for HCV. In general, patients tested in the ED and inpatient setting had more barriers to successful linkage, including increased homelessness and co-morbidities.

Due to the significant differences in the demographics of patients that tested positive in each site, it is not surprising to see that the testing sites also experienced differences in eligibility for linkage, reasons not linked, and proportion linked to care. GIM, CID, and FM all had much higher percentages of HCV RNA+ patients that had a need for linkage to an intake appointment compared to the ED and Inpatient sites. Among those that were considered to have a need for linkage, GIM, CID, and FM also saw higher percentages of linkage to HCV care when compared to the ED and Inpatient sites. By way of explanation, we hypothesize that patients currently in care at an outpatient clinic are more likely to engage in future follow-up appointments, such as an HCV intake appointment. Engaging in regular medical care, as well as building an established relationship with a healthcare provider, helps to cultivate trust and motivation to continue following and adhering to medical guidance, such as attending a follow-up appointment. The Infectious Disease clinic, unsurprisingly, had a high rate of patients with co-morbid infections, but a high linkage percentage, likely due to the established relationship.

The ED has proven to be an excellent testing site for identifying new HCV-infected patients. However, for patients diagnosed in that arena there are formidable barriers to linkage including homelessness, IDU, and co-morbid conditions. This does not necessarily imply that the ED should not be involved in HCV screening. In fact, our data point to the opposite interpretation. There are many patients at this medical center that were identified in the ED that might not have been captured in other another setting. And even though the screening criteria were broader in the ED than other sites, the disease rate was extremely high, with 7.2% of patients screened found to be RNA+. The data does, however, suggest that additional linkage strategies are necessary to successfully link ED patients to care. The current navigation system, which is very effective in other parts of the medical center, has not demonstrated itself to be sufficient for linking ED patients to care. Both the ED and inpatient sites show high percentages of patients being unreachable/lost to follow up, as well as having no contact information on file. It is conceivable that there is a rate of linkage threshold, below which it is not beneficial to implement HCV screening. However, there is not acceptance in the scientific community at what this rate is, and the modest linkage success rate of 22.6% (from the ED, of all patients RNA+) still translates to 224 patients over a 2 year period that were linked to HCV care. Ultimately almost as many patients were linked to care from the ED in the study period as patients from the GIM clinic (224 from the ED compared to 269 from GIM).

This data also reveals important points regarding treatment success. For all sites of diagnosis, a fairly high portion of those patients that were linked to care actually started treatment for HCV, from a low of 32% of those diagnosed in CID to a high of 54% from GIM. Overall, this is higher than rates identified in other settings [15, 20]. It is thought that this is due to an overall high capacity and high commitment to treating HCV at our institution. There was also a high level of treatment adherence, regardless of the initial site of diagnosis, from a low of 40% of patients diagnosed Inpatient to a high of 100% diagnosed in CID. Rates of medication adherence vary across the literature, and we suspect the excellent rates achieved at our institution are due to high case management capacity at our institution, which helps retain patients in care. Differences in treatment success across sites of diagnosis may reflect differences in levels of scrutiny toward medication starts dependent on patient co-morbidities and psychosocial factors.

Finally, one notable demographic finding that held across most testing sites and deserves mention was that white patients were significantly more likely to be Ab+ and RNA+ than patients in other racial or ethnic groups. This is not reflective of the institution’s overall demographics, but is consistent with the trend noted throughout Massachusetts of white patients being disproportionately affected by HCV, and is likely a feature of the opioid epidemic in our area [21].

As this program continues, there are several strategies we hope to implement to improve linkage and treatment rates across all sites, especially in the ED and inpatient sites. These strategies will be tailored to each site in order to appropriately address the different barriers encountered.

### Limitations

This evaluation has some limitations. First, this represents data from a single center in an inner city with a high underlying disease burden, and findings may not be similar at other institutions. The HCV screening algorithm is different in each location at the medical center, and this may account for some of the differences in the patient populations diagnosed with HCV at different sites. The distinctive screening strategies were a result of the real-world difficulty of obtaining support to implement large-scale screening at separate clinics, with diverse stakeholders and diverse staffing patterns. However, the main difference in the screening algorithm is whether or not patients outside the birth cohort are eligible for testing. The other differences encountered, including race/ethnicity, homelessness, and co-morbid conditions are not explained by the testing algorithm. Information technology limitations precluded the ability to obtain certain data that were of interest, including the number of patients that were seen in multiple settings and the number of times the BPA fired but wasn’t selected at each site, so it was not possible to obtain data on the true eligible patient population at each site. Finally, Massachusetts has had an individual mandate for health insurance since 2006, and that will continue after the federal mandate ends in 2018. Approximately 97% of state residents have health insurance, the highest in the nation [22]. The state Medicaid program also covers medications for those with chronic HCV without restrictions related to liver damage, sobriety and prescriber specialty. Insurance is therefore significantly less of a barrier due to state regulations than it might be in other states.

## Conclusion

Our data show that at each of five testing sites that implemented an HCV screening and linkage to care program at one medical center, there were differences in the sociodemographic characteristics of patients diagnosed with HCV, as well as differences in the success linking patients to outpatient care. They were differences in many key drivers of success at each stage of the HCV care cascade, based on where they initially presented to the HCV program. Understanding these differences is essential to making an informed decision about in which environment health-care systems should focus resources for HCV screening and linkage to care efforts. At this medical center, the ED was the site that offered the most opportunity for screening patients, identified the largest number of HCV cases, and had among the poorest linkage success. The GIM clinic screened fewer patients and identified fewer RNA+ cases, but ultimately had a higher number of patients linked and treated. The answer of where to focus resources may be different for every institution, but this analysis can inform conversations about resource use.

## Supporting information

S1 Dataset Antibody, RNA, and linkage data set. (XLSX)
